# Stakeholder acceptability of the ROWTATE vocational rehabilitation intervention in England: an interview study

**DOI:** 10.1136/bmjopen-2024-098048

**Published:** 2025-10-23

**Authors:** Claire Mann, Rebecca Lindley, Denise Kendrick, Kathryn A Radford, Jain Holmes, Blerina Kellezi, Roshan das Nair, Steve Fallon, Stephen Timmons

**Affiliations:** 1 University of Nottingham - University Park Campus, Nottingham, UK; 2 Centre for Academic Primary Care, University of Nottingham, Nottingham, UK; 3 University of Nottingham, Nottingham, UK; 4 Medical School Queen’s Medical Centre, Nottingham, UK; 5 Division of Primary care, School of Medicine, Nottingham University, Nottingham, UK; 6 Institute of Mental Health, University of Nottingham, Nottingham, UK; 7 Centre for Academic Primary Care, Lifespan and Population Health, University of Nottingham, Nottingham, UK

**Keywords:** REHABILITATION MEDICINE, OCCUPATIONAL & INDUSTRIAL MEDICINE, Psychosocial Intervention, Trauma management

## Abstract

**Objectives:**

The ROWTATE intervention helps people experiencing trauma to return to work (RTW) through vocational rehabilitation (VR) support from occupational therapists (OTs) and clinical psychologists (CPs). This study aims to explore and understand the acceptability of VR after traumatic injury for patients, therapists and employers.

**Design and setting:**

Qualitative interviews in eight major trauma regions, UK.

**Participants:**

Interviews were undertaken with a range of stakeholders—15 patients, 15 therapists and 6 employers. Data were analysed using the theoretical framework of acceptability.

**Results:**

Stakeholders understood the aim of the intervention was to support people to RTW and perceived it as effective in achieving this. Patients and therapists understood the benefits of working with a combination of occupational therapy and clinical psychology. The intervention fits with the values of patients wanting to recover, therapists wanting to offer support and line managers wanting to meet employer and employee needs.

Patients reported they could not have achieved RTW without the intervention, and their therapist helped them feel less alone. Therapists felt that their work was rewarding, effective and had good outcomes. Patients perceived remote delivery as less burdensome than attending in person. Therapists felt they wasted time on non-patient activity, such as (re-)arranging appointments.

Employers discussed the difficulty of balancing employer and employee needs and managing uncertainty. Some workplace policies lacked flexibility, and without the ROWTATE intervention, employers lacked confidence in supporting employees RTW.

**Conclusions:**

A VR intervention delivered remotely by OTs and CPs is acceptable to patients, therapists and employers.

**Trial registration number:**

ISRCTN43115471.

**STRENGTHS AND LIMITATIONS OF THIS STUDY**

- A diverse range of stakeholders ensures that multiple viewpoints are considered.
- Semi-structured qualitative interviews allow for in-depth exploration of experiences.
- Analysis using a structured theoretical framework for acceptability allows for a systematic assessment of factors influencing the intervention’s acceptability and adds rigour to the analysis.
- Some selection bias may be present, resulting from a lack of representation of disengaged patients and fewer employers than initially planned.
- Participants may have held more positive views of vocational rehabilitation (VR) than those not willing to be interviewed, and it is possible that our findings may not fully represent the views of a broader diversity of patients, therapists and employers involved in VR.

## Introduction

The ROWTATE intervention helps people experiencing trauma to return to work through vocational rehabilitation support from occupational therapists (OTs) and clinical psychologists (CPs). This research explores the acceptability of ROWTATE to patients, therapists and employers.

There are at least 45 000 individuals in the UK who experience severe or major traumatic injury each year and over 5 000 000 individuals have less severe trauma.^1^ One third of patients have not returned to work 12 months after injury,^2^ and injuries result in almost 29 million working days lost in the UK each year.^3^ Returning to work is a key rehabilitation goal for many trauma patients in terms of financial well-being, personal identity, self-esteem and helping to meet psychosocial needs.^4^ Retirement due to disability from injury places an additional burden on society, compounding the existing challenges of an ageing population and workforce.^5^ As well as the economic and societal burden, early retirement negates the physical, social and psychological benefits of working for the individual.^6^ Despite this, there is limited access to vocational rehabilitation (VR) post-injury in the UK NHS.^7^

The ROWTATE VR intervention (www.rowtate.org.uk) was designed to meet the VR needs of patients after traumatic injury and support return to work (RTW). The remotely delivered intervention was individually tailored, and support was provided by occupational therapists (OTs) and, where needed, clinical psychologists (CPs) for up to 12 months, dependent on need.^8^ A more detailed description of the intervention and its development can be found elsewhere.^9^ The ROWTATE VR intervention is being tested within a randomised controlled trial (RCT) and process evaluation, which uses a range of Implementation Science tools to understand factors affecting the processes and the results of implementation of the intervention.^9^ Acceptability is one Implementation Science tool used to understand stakeholder perceptions about how agreeable intervention components are.^10^ The following table outlines the ROWTATE intervention key components and activities:

Acceptability of complex interventions is multifaceted and includes perceptions of both those delivering (healthcare professionals) and receiving the intervention (patients and employers).^11 12^ From the perspective of intervention recipients, if an intervention is acceptable, patients are more likely to adhere to what is offered and benefit from it, leading to positive health outcomes.^11^,^13^

There is a small number of studies exploring the acceptability of VR interventions.^14 15^ Most research published in the last decade focuses on specific groups, such as veterans^16^ or specific conditions such as stroke^17 18^ or multiple sclerosis.^19^ Building on this, our previous acceptability study undertaken during feasibility testing of the ROWTATE intervention^20^ demonstrated that the ROWTATE VR intervention is acceptable to patients and therapists and identified a number of barriers and facilitators of acceptability.

This paper explores the acceptability of a remotely delivered VR intervention provided to a diverse group of trauma patients. This offers the opportunity to consider the acceptability of VR to a broader population than previous studies and adds to the literature on the acceptability from the deliverer (from multiple professions) and recipient perspectives.

## Method

This qualitative study was undertaken in the UK with patients admitted to eight major trauma centres who received the ROWTATE VR intervention, therapists (OTs and CPs) who delivered the intervention, and employers of both patients who received the intervention and those who did not. Each participant undertook a phone or online interview with a researcher. The ROWTATE intervention is described in our protocol paper^8^ and table 1.

**Table 1:** Components of the ROWTATE intervention and delivery mechanisms

The ROWTATE intervention commences within the first 2 weeks of injury. It is individually tailored to the patient’s need and employment context in terms of content, dose, intensity and duration (up to 12 months). The intervention includes:	
**Component**	**Activities**
Assessing the impact of the injury on the participant, family and the participant’s role as a worker	Occupational therapist (OT) assesses the impact of injury on the ability to return to work (RTW), using observation and standardised functional assessments, for example, Job Demands Analysis, Functional Capacity Evaluation. Worksite assessment. OT gathers reports of standardised assessments completed by other members of the healthcare team, for example, mobility, cognition and communication assessments. OT completes job analysis through discussion with the patient, obtaining a job description and the use of published job dictionaries.
Setting and reviewing vocational goals	OT and CP set goals in line with patient needs and where OT/CP joint working develops a case formulation. Goal attainment scaling may be used.
Educating participants, employers and families about the effects of the injury and its impact on work, and finding acceptable strategies to lessen that impact	OT/CP provides ongoing education and advice to the patient, family and employer. Employer engagement.
Monitoring and adjusting the participant’s post-injury life and work goals	OT provides feedback on performance and monitors RTW to ensure stability. Supported by CP where necessary.
Preparing participants for work by establishing structured routines with gradually increased activity levels to build work tolerance and opportunity to practise work skills	OT/CP deliver flexible, individually tailored rehabilitation to support RTW, including work preparation, RTW planning, work hardening programmes, negotiating phased RTW and workplace adjustments with employers.
Liaising with relevant stakeholders such as employers, employment advisors (eg, occupational health), solicitors and the healthcare team to advise about the effects of the injury and to plan and monitor a phased return to work (RTW)	OT case coordinates patient’s RTW rehabilitation across all sectors, engaging with employer to negotiate and facilitate RTW. OT communicates openly in writing with stakeholders about work performance during RTW process.
Routine monitoring of mood and emotional issues, via routine use of questionnaires, observation and responses during clinical sessions, by the OT	At initial assessment and again at 6 months post-injury, OT screens participants for mental health problems using standardised psychological measures (GAD-2, Whooley Depression questions, Hospital Anxiety and Depression Scale, Impact of Event Scale, PHQ Panic Disorder Questionnaire). If participant scores within the ‘case’ or ‘borderline’ threshold for any measure, the OT refers to the CP for further assessment.
Discussion with or referral to a CP where needed. The CP will deliver individualised psychological assessment and/or treatment and work with the OT to facilitate the patient’s RTW	OT considers results of psychological testing and/or clinical observation and makes a referral to clinical psychologist (CP). Following assessment, CP may (1) Identify no need and recommend no intervention, (2) Advise the OT to monitor the participant for a month then re-screen, (3) Refer the participant to other local CP or mental health services or (4) Deliver ROWTATE intervention to the participant. Psychological interventions delivered 1–1 include evidence-based approaches for managing trauma-related mental health issues such as anxiety, depression and post-traumatic stress disorder, assessment of the impact of mental health problems on work ability, teaching coping strategies, for example, fatigue and anxiety management for use in the workplace. OT and CP operate as a multi-disciplinary team to support patient needs. Meet patients individually and together where beneficial. Meet together to discuss patient and actions.
Delivery up to 12 months	Patient is discharged when returned to work, work is stable and no longer requires monitoring or support or at 12 months, whichever is sooner.

OT, Occupational therapist; RTW, Return to work.

### Ethics

This study received ethical approval from the North of Scotland Research Ethics Committee (REC reference 20/NS/0140). All procedures involving human participants were conducted in accordance with the ethical standards of the institutional and national research committee and with the 1964 Helsinki Declaration and its later amendments or comparable ethical standards.

Informed consent was obtained from all individual participants included in the study. Participants were provided with detailed information about the study’s purpose, procedures, risks and benefits, and were given the opportunity to ask questions before providing written consent.

### Public and patient involvement (PPI)

Patient and public involvement (PPI) supported designing the interview schedule and conducting the research.

### Inclusion criteria and recruitment

We planned to interview 15 patients, 15 therapists and eight employers, the sample size based on the concept of information power.^21^ Patients and therapists participating in the RCT were invited to express interest, and those who did were given an invitation pack including information and consent forms. Patients were purposively sampled to represent a range of sites, ages, genders and injury severity. Therapists were purposively sampled to represent a range of roles, genders and location/type of role. We initially planned to interview employers of patients who had experienced the ROWTATE intervention, but a low number of patients gave permission to contact their employers. We therefore expanded inclusion criteria to employers with experience of managing, representing or supporting people with disabilities. We approached our local employer network (contacts of the research team and our Business School) and approached employers by email, inviting expressions of interest and responding to these with information and consent forms. All participants gave informed consent.

### Data collection

Interviews were conducted by four experienced qualitative researchers (including CD, RL, CM, BK) and one PPI group member (SF), who was trained in interviewing and jointly interviewed some patient participants alongside a researcher. Interviews were conducted between March 2023 and September 2024. The interview schedule was created based on our feasibility study^20^ and the Theoretical Framework of Acceptability (TFA),^12^ which reflects the multifaceted concept of acceptability across seven domains: affective attitude, burden, intervention coherence, ethicality, opportunity costs, perceived effectiveness and self-efficacy. Participants were asked about their experience of the intervention and its acceptability, across all domains in the TFA.^12^

All participants were given an information sheet and consent form at the time of booking the interview, and gave consent in writing before or verbally during the interview. Interviews were undertaken by telephone or online, depending on participant preference. Interviews were recorded and transcribed verbatim either by the University transcription service or automated transcription, then checked, corrected and anonymised by the research team and stored securely.

### Data analysis

Anonymised data were transferred to the software package NVivo V.15. A framework analysis approach was undertaken.^22^ Data were coded into the domains of the TFA by one researcher (CM). To ensure reliability, a second researcher (RL) independently double-coded 10% of the transcripts. Coding discrepancies were discussed and resolved through consensus, with agreed criteria recorded in a coding decision log. A working analytical framework was developed and applied across all transcripts. Data were then charted into a framework matrix to facilitate comparison across cases and domains. A final table of all analysed data was refined through iterative discussion with the research team to a summary table of key data underpinning each domain sub-theme. A representative summary of this table is given in the online supplemental data S1.

## Results

We interviewed 15 patients, 15 therapists and six employers. Participant characteristics are shown in tables2^4^.

**Table 2:** Patient stakeholder characteristics

Patient characteristics (n=15)	
Gender	Male: 9 (60%) Female: 6 (40%)
Age	Range: 22–71 (mean: 52.36, median: 58.5)
Marital status	Single: 5 (33%) Married/cohabiting: 8 (53%) Long term relationship: 1 (7 %) Separated: 1 (7%)
Years of education	Range: 11–20 (mean: 14.43, median: 14.00)
Injury	Fractures: 13 (86%) Head injury: 1 (7%) Nerve: 1 (7%) Other: 9 (64%)
Injury severity	9–15: 12 (80%) >15: 3 (20%)
Injury type	Fall: 7 (47%) Road Traffic Accident (RTA): 7 (47%) Other: 1 (7%)
Injury location	Home: 5 (33%) Road: 8 (53%) Work: 0 Educational establishment: 0 Other (boat, street): 2 (14%)
Employment	Employed: 11 (73%) Self-employed: 4 (27%)
Time from injury to interview	Range: 4–20 months (mean: 13.43 months, median: 15 months)
Involvement	CP involvement: 5 (33%) Employer involvement: 7 (47%)
Returned to work	13 (87%)

CP, clinical psychologist.

**Table 3:** Therapist stakeholder characteristics

Therapist table (n=15)	
Gender	Male: 2 (13%) Female: 13 (87%)
OT/CP	OT: 12 (80%) CP: 3 (20%)

CP, clinical psychologist; OT, occupational therapist.

**Table 4:** Employer stakeholder characteristics

Employer (n=6)	
Gender	Male: 2 (13%) Female: 13 (87%)
Ethnicity	White British: (100%)
Size of business (S<50, M=50 to 250, L=350+ employees)	Small: 1 (17%) Medium: 2 (33%) Large: 3 (50%)
Type of business	Freight: 1 (17%) Banking: 1 (17%) Financial services: 1 (17%) Electrical: 1 (17%) Rail: 1 (17%) Consultancy: 1 (17%)
Employer of ROWTATE participant	Yes: 3 (50%) No: 3 (50%)

### Acceptability data

#### Affective domain

The affective domain considers attitudes towards the intervention.

ROWTATE helped patients feel less alone and supported when they might not otherwise have been supported by NHS services. Many patients felt they could not have achieved recovery or RTW without the intervention.

Excellent, invaluable, necessary, without it I would’ve failed; and just incredibly important… I think it’s incredibly important that everybody gets the support that I got, because you would just fail without it (PA02)

ROWTATE offered an opportunity for therapists to help a broader range of clients than normal, and they felt that their work was rewarding, effective and had good outcomes.

I think it’s just really nice to be part of something that seems to make a difference… it definitely seems to be helping the people I’m working with (TA02)

Employers felt that ROWTATE offered useful support for employees, which helped them to feel more supported and less alone during the rehabilitation process. All employers suggested that ROWTATE should continue to be offered (after the research programme) to people after injury to help them to RTW.

The support that they’ve offered to us as an organisation, and also, the individual coming back in, I think it’s been fantastic (EMP06)

#### Burden

The burden domain considers the perceived amount of effort that is required to participate in the intervention.

Patients perceived minimal burden from participating in ROWTATE, and appointments and rehabilitation sessions/activities were seen as a positive investment of time.

I was completely available because I wasn’t working, and I didn’t have anything else to do. It felt effortless because of (the therapist’s) approach. There was an investment in terms of how much time I needed to put in on working on those targets, but obviously, that’s expected (PA02)

For therapists, time was the biggest burden, especially with non-patient-facing activities such as arranging appointments and record keeping. Where therapists could work flexibly to suit their needs, this eased the burden and facilitated time-management.

The time it took to constantly be calling them or setting aside time to arrange an assessment with them, and them not being available. Or setting aside time to do the assessment and being interrupted by someone else on the hospital ward. Or just not contacting me… That was just very time consuming (TA01)

The employers in the study did not perceive any burden from the intervention.

#### Ethicality

The ethicality domain considers the extent to which the intervention has a good fit with an individual’s value system.

Patients felt the intervention was a good fit with their values as the support was responsive, not intrusive and helped patients feel ownership of actions and progress.

You know, I’m very determined, and I needed to find the strong bit, and she (OT) helped me to locate that (PA07)

Therapists saw the intervention as providing a safety net supporting patient self-efficacy and leading to positive outcomes, and this fit with the therapists’ ethical aims to make a difference and help people.

I think it definitely fits with my values of how I can support people long-term (TA13)

Therapists mentioned their role was a positive counter to the push (by litigators and insurers) for patients to stay off work to increase their financial claim.

Employers suggested the intervention aligned with their desire to encourage and support employees back to work at the right time and helped them to balance employee and organisation needs.

I think it wholly fits with the values of our organisation. It provides an extra level of care for employees, and it helps the organisation think about that employee’s journey back to work in the right way and the healthiest way for the employee (EMP06)

Employers felt the intervention could be perceived as an employee benefit alongside private health provisions.

#### Intervention coherence

The intervention coherence domain considers the extent to which the participant understands the intervention and how it works.

Stakeholders all displayed a clear understanding of ROWTATE based on its unique ability to support people to RTW and meaningful activities, to facilitate recovery through physical and mental health support and to facilitate an ongoing network of support, including employers and line managers.

Patients understood the aim of the intervention was to support them to RTW, or others to meaningful activity, and understood the intervention was personalised to meet their needs and refer or signpost them to other services where relevant.

I guess it is driven, tailored to your role, and so she gave loads of support, but she didn’t sort of say, I advise you to do this, that the other, she sort of just gave me the options and let me choose from them (PA12)

While patients had a generic understanding of rehabilitation and recovery, for therapists, the intervention was specifically mapped to VR.

I think helping people get back to work after an injury is what it is. That was the focus of my mind throughout it (TA13)

Both therapists and patients understood ROWTATE filled a gap in usual care. Lack of engagement was disheartening and time-consuming for therapists. Some patients did not have the financial or social capacity to engage or did not engage because they felt they did not need support. Disengagement could also be in part due to the recruitment of patients who did not need this specialised support (rapidly healing injuries, lack of cognitive injuries). This will be explored further as part of a broader trial evaluation.

The intervention supported employers in dealing with the uncertainty surrounding recovery. Employers also understood that the intervention supported people to RTW and perceived this to be effective.

I met with the OT and the employee on a relatively frequent basis. So once a month or … when things changed with the employee’s situation, and that gave instant access for us all to talk very pragmatically and openly about the situation, the working environment and what was possible and what wasn't (EMP06)

Both patients and therapists understood the benefits of a personalised approach using occupational therapy and clinical psychology.

Part of the problem was actually in the mind as well as the body… So, the whole engagement with ROWTATE was really essential for me, because I don’t think I would have …got back to work in a sustained manner, if I hadn’t had the ROWTATE programme (PA14)

I think the whole principles of the ROWTATE study, and the joint working with psychology…in my team anyway, we were certainly aware of the importance of the psychology support and joint working, but it just doesn’t happen. So hopefully the study will highlight the massive benefits, and that really VR can only work with the psychology side by side (TA15)

Where employers were involved with the intervention and had direct contact with their employee and OT/CP, it was perceived as helpful by all parties; however, some patients did not consent to their OT/CP liaising with their employer.

#### Opportunity cost

The opportunity cost domain considers the extent to which benefits, profits or values must be given up to engage in the intervention.

For patients, the only cost was time, but ROWTATs were considered a valuable use of their time. The remote delivery of the intervention reduced potential travel costs and time for most participants.

For therapists, time was also the most important cost, and time lost trying to communicate with and engage with patients instead of providing services was an opportunity cost. The remote delivery of the intervention reduced the potential cost of travel and time; however, it took longer to build rapport, and therapists felt patients were more likely to cancel appointments than if they had been seen in person.

For employers, the uncertainty around time to recover caused the biggest cost, as it made it difficult for them to plan.

#### Perceived effectiveness

The perceived effectiveness domain considers the extent to which the intervention is perceived as likely to achieve its purpose.

Patients perceived ROWTATE was effective in helping people RTW and positively impacted their overall recovery journey.

It was really, really pivotal in getting my confidence back and in not despairing… she was really helpful in saying, ‘Yes, this is normal. You’re not going bonkers that you’re finding this difficult or that you’re tired or that you’re sad or that you can’t cope with people,’ (PA06)

Having both occupational and psychological therapy when needed was perceived as key in this rehabilitation. For some, the intervention helped plug a gap in usual services where there was no support for RTW after serious injuries. The contact that started early and lasted for up to a year offered continuity for patients to seek reassurance, where they might have otherwise had multiple General Practice (GP) appointments.

Otherwise, I would have been contacting my GP, because I wasn’t going as fast as I thought I was going…if I hadn’t spoken to (TO24), I wouldn’t have had that knowledge, and therefore I’d be using more of the NHS time, which was probably unnecessary (PA14)

Being able to receive the intervention remotely meant that some patients accessed it more easily, wherever they happened to be, and because travel for in-person appointments was not needed, even patients who could not travel benefited.

Some patients perceived the intervention as effective when it engaged both employer and patient, but others also felt it was effective when therapists advised the employer about RTW vicariously via the patient.

With the help of my OT, I was able to have those conversations with my manager. She got involved in a three-way conversation too, and we sort of looked at my working pattern. We immediately stopped the overnight travel, and we tried to minimise as much of the day-to-day travel as possible (PA12)

Some participants reflected that the intervention was effective even when there had been no contact between the therapist and the employer. Some patients felt the intervention was more effective at supporting RTW than their own organisation’s occupational health service, which provided minimal direct support to employees.

Therapists perceived ROWTATE as effective in RTW and sustaining employment.

One thing I found quite successful is having that regular contact with someone after they’ve gone back to work… and making sure it’s still all going okay… I’ve had some participants who’ve said, ‘Because I look okay, people are just dumping loads of work on me. But actually, I’m really struggling’. Then I’ve been able to have conversations with the employer, or I’ve been able to give them advice about how they manage it, and that’s helped (TA09)

Therapists perceived the intervention to be effective in supporting employers, and in particular, smaller organisations otherwise unable to access relevant support, for example, occupational health services.

I’ve had direct feedback … that this has been really useful, and it’s helped because there’s a lot of uncertainty on that side. Employers generally… (have) very positive intentions about wanting to support people back into the workplace, but a lot of concern about how actually to do that. And it’s probably quite hard to express that when you’re in a management position or an HR position. Obviously, they’re often looking for independent advice anyway, going out to independent medics for occupational health advice, etc, so that’s another place where we can feed in (TA11)

Employers perceived the intervention as providing achievable, practical advice on supporting employees and explaining their needs, helping people to RTW at the right time.

We have benefited from the service that ROWTATE provides. With the unfortunate circumstances … of our senior leadership team that is responsible for driving the future of our organisation. And the support that she has had, and I received in terms of that RTW process, it’s been a success frankly, because she’s still here now and back to full-time and enjoying herself, or at least she tells me she is. So that is a success and honestly, (…) this time last year, I couldn’t have had on my heart have said that was going to be the case, because of course you start to have doubts when someone has been off for that period of time (EMP01)

Employers perceived the holistic multidisciplinary support provided with continuity was effective, although some suggested it was needed longer than provided (when support ended at 12 months).

#### Self-efficacy

The self-efficacy domain considers the participant’s confidence that they can perform the behaviour(s) required to participate in the intervention.

Most patients suggested they were confident to participate in the intervention and creating shared goals with the therapist and employers gave patients’ confidence to achieve their aims.

I felt confident. I guess it depends on how they deliver it to you, or what your personality is, or a bit of everything. I felt confident that I could access it. I think I probably wouldn’t have had the initiative or known how to look for some of it otherwise (PA04)

Remote participation was seen as easy, and even first-time users succeeded in connecting online. Some therapists were nervous about delivering the intervention remotely, but with time, training and mentoring support, therapists felt confident delivering the intervention to patients and employers.

I think you guys did a good training, but there’s nothing like actually learning when you’re doing the job. So, there’s been a lot of things where I’ve gone away when I’ve been with a participant and gone, okay, I’m going to need to take some time to get my head around this, or to do some research, or to do some extra learning, and you’re not going to know what that is until you’ve been with that participant. It’s just experience, isn’t it? (TA08)

Positive feedback from patients, building relationships and seeing outcomes helped to build confidence. Many therapists suggested they felt they developed new skills and greater confidence from participating in the intervention.

Employers reported they were willing to support employees but lacked confidence in identifying and deploying the right RTW plan because they had not previously supported someone in this situation.

Thankfully, in my 20-year career, this is the first time I’ve ever dealt with a severe injury like this. So, I take the assumption, and I may be wrong, that the prevalence of these sorts of injuries is low, but of course the impact of them is going to be huge (EMP01)

Employers trusted the advice given by therapists, seeing them as experts, and were confident to follow that advice, but suggested that some workplace sickness and absence policies lacked flexibility, which impacted their confidence to provide the support required.

## Discussion

### Summary

All stakeholders understood that the aim of the intervention was to support people back into work and to stay in work, and perceived it as effective in achieving this aim. Patients and therapists understood the benefits of working with a combination of occupational therapy and clinical psychology. The intervention fits with the values of patients wanting to recover, therapists wanting to offer support and line managers wanting to meet the needs of their employer and the employee.

Patients reported they could not have achieved RTW without the intervention, and their therapist helped them feel less alone. Therapists felt that their work was rewarding, effective and had good outcomes. Patients perceived remote delivery as less burdensome than attending in person. Therapists felt they spent a lot of wasted time on non-patient activity, such as (re-)arranging appointments.

Employers discussed the difficulty of balancing employer and employee needs and managing uncertainty. Some workplace policies lacked flexibility, and without the ROWTATE intervention, employers lacked confidence in supporting employees RTW.

Patients and therapists perceived ROWTATE as effective in helping people RTW at the right time. Therapists and employers perceived the intervention to be effective in supporting employers to help people integrate back into work. In conclusion, the study demonstrates that the ROWTATE intervention is acceptable to stakeholders.

### Comparisons with other literature

Patients and therapists perceived the ROWTATE VR intervention as an effective way of combining support from OTs and CPs to help people go back to work. This important finding fits with evidence about the importance of the biopsychosocial perspective for RTW and the benefits of both physical and psychological rehabilitation shown in studies of particular populations.^16^,^1923^ In our study, patients reported strong positive feelings about the intervention, helping them to feel less alone and supported in their recovery journey. This is important given previous evidence on loneliness impacting the rehabilitation journey.^23^,^27^

The intervention fit with the therapists’ professional values to make a difference and help people, a known factor impacting the acceptability of health interventions and successful outcomes.^28^ However, our research showed that lack of engagement was disheartening and time-consuming and an opportunity cost for therapists. This is an important finding that other studies identify as a risk to therapist retention^29^ and potentially having a negative impact on acceptability.

Only 28% of UK employers may have support such as occupational health services,^30^ and ROWTATE helped fill this gap. Employers perceived the intervention as effective at providing practical advice on how to support employees by clearly explaining their needs as patient. This is consistent with a small number of studies which engaged with line managers involved in supporting patients.^31^ Our findings fit with the existing work that suggests employers are generally positive about RTW and the support of VR.^17^ Employers in our study suggested the intervention helped them learn more about rehabilitation and how to support individual employee needs, and this is consistent with one study about the perspectives of employers involved in rehabilitation, which highlighted a key theme of ‘empowerment through knowledge’.^18^ Uncertainty was the biggest cost for employers as it made it difficult for them to make plans, such as recruiting temporary staff to cover absence, also found in other studies.^1831^,^33^ Employers also suggested that RTW procedures lacked flexibility and policies were rigid, which impacted their confidence to provide the support required and participate in the intervention. One study exploring employer dilemmas in RTW suggested that some policies do not always adequately prompt employers to engage in RTW,^34^ and one review of ethical perspectives relating to RTW identified that individual concerns do not always align with organisational (and sometimes societal) values.^28^ Employers felt that ROWTATE provided support for their employees that was not on offer anywhere else, and this could be the reason for the positive findings of both our research and the wider literature.^16^,^18^

### Strengths and limitations

The study includes a diverse range of stakeholders, ensuring that multiple viewpoints are considered, enhancing the richness of the data. Semi-structured qualitative interviews allow for in-depth exploration of experiences. The application of a structured theoretical framework for acceptability, allowing for a systematic assessment of factors influencing the intervention’s acceptability, adds rigour to the analysis.

Some selection bias may be present in our study, resulting from a lack of representation of disengaged patients and fewer employers than initially planned, only some of whom had experienced the ROWTATE intervention with an employee. In addition, those willing to be interviewed may have held more positive views of VR than those not willing to be interviewed. Although we undertook 36 interviews, it is possible that our findings may not fully represent the views of a broader diversity of patients, therapists and employers involved in VR.

### Implications for research/practice

Our study has shown that the ROWTATE intervention is acceptable to stakeholders. If the ROWTATE RCT demonstrates effectiveness, the VR intervention should be widely rolled out. On wider roll-out, the intervention needs to be delivered as it was during the ROWTATE RCT to maintain the high levels of acceptability found in our study. Policy frameworks that support its implementation, including guidelines and funding mechanisms that encourage adoption, will be needed. Ongoing evaluation of acceptability to all stakeholders is important to identify emerging issues and allow for timely adjustments. Roll-out would require an implementation plan involving strategies and evaluation measures to assess those strategies.

Employers play a crucial role in the VR process, and more research is needed exploring different approaches to engaging employers in the VR process and also in the development and implementation of VR programmes going forward.

## Supplementary material

10.1136/bmjopen-2024-098048 online supplemental file 1

## Data Availability

Data are available upon reasonable request.
