# Supplementary material for: Stakeholder acceptability of the ROWTATE vocational rehabilitation intervention in England: an interview study
Source: BMJ Open. 2025 Oct 23;15(10):e098048. doi: 10.1136/bmjopen-2024-098048 (PMC12557785; doi:10.1136/bmjopen-2024-098048)
Supplement: online supplemental file 1 [file bmjopen-15-10-s001.pdf]

Acceptability Analysis data examples by domain and participant group

Acceptability domain	Patients voice	Therapist voice	Employer voice
**Affective**<br><br>***How an individual feels about the ROWTATE intervention***	It really is like a comfort blanket that you have something you can cling to whilst you are getting better. You know that ten o'clock on a Tuesday or Wednesday morning [TO22] will be phoning, if you have any issues you could ask her about it. (PA05)<br><br>I could let off those difficult thoughts so that I could then proceeds, so it was very different from that, you know, very gently letting me go forward rather than backwards, so I always looked forward to it. I can't say – when I spoke to the psychologist I was very low so I don't think – looking forward is the wrong word, but it sort of felt necessary and helpful (PA06)<br><br>I wouldn't be in the position where I am now I don't think if I wasn't given that support, because there was just no way that I could have ever - I could have never of thought of the things that she told me to do by myself, (PA11)	I've been quite happy with it. It's been lovely to see how well people are progressing when they've got the right support in place. (TA09)<br><br>I think it's a really rewarding job, doing the ROWTATE stuff, because you do get those good outcomes of getting people back to work. (TA10)<br><br>I used to get anxious about having a session, it just felt a bit funny, but now I feel a lot more settled, a lot more comfortable, and I think it's a really nice (TA13)	I felt like I learned a lot, didn't do a very good job frankly, but learnt a lot in the meantime. (EMP01)<br><br>It's providing that specialist support ultimately for the employee who's going through that traumatic situation and is fearful for their life and their livelihood, it's something positive for them at a time where they perhaps need an extra level of support rather than a one hour consultation with somebody and then they're wondering in the dark what's happening for the next X weeks or months, that's good. (EMP04)<br><br>I think the level of service provided was over and above any expectation that I've ever had…and so all of it was of key value to me and the decisions that that I made across that year. (EMP06)

Acceptability Analysis data examples by domain and participant group

**Burden**<br><br>***The perceived amount of effort required to participate in the ROWTATE intervention***	One of my targets was to build my physical capability and so then that was jogging or walking for an hour, I'd built it up to an hour which I was really pleased with. I wasn't set an hour target, I was just set ten minutes, and as I was able to do more I continued, because I felt that sense of achievement and then the exercises that she would email me, they'd take maybe an hour, depending how many she sent me, an hour, an hour and a half to two hours, but they wouldn't, they wouldn't be done all at once, because of the concentration level, so that would be spread out throughout, and then building concentration in terms of reading, I initially was only doing ten minutes a day, but then was building it up to do a couple of hours a day. So yeah there is quite time investment, but not until you're ready (PA02)<br><br>It was no effort at all, I just, I'd look forward, because obviously I wasn't sleeping, I was here and my husband gave me the support, my family gave support, but my husband worked, you know, he took time off but I was here by myself so it became like looking forward to my weekly meeting. (PA11)<br><br>I felt it was just right for me. What I felt was that I was offered as much as I needed (PA12)<br><br>She would always push me, not too hard, but equally push me (PA14)	The time it took to constantly be calling them or setting aside time to arrange an assessment with them and them not being available. Or setting aside time to do the assessment and being interrupted by someone else on the hospital ward. Or just not contacting me. And telling me that everything was going to be really difficult but then disengaging. That was just very time consuming. (TA01)<br><br>Financially it's had no impact on me. I've not had any, had to spend any money, I think it's because I'm delivering stuff from work. I have used my, so sometimes I do work from home and I have used my mobile phone to get in touch with patients, but I just, I use, because I don't have a work mobile phone, I just like withhold my number on that, because obviously I don't want patients to have my personal mopbile (TA03)<br><br>It is quite a lot to have to record everything that you do. But I can see the importance of that for the purposes of the research study. (TA04)	No data identified in initial coding

Acceptability Analysis data examples by domain and participant group

**Ethicality**<br><br>***The extent to which the ROWTATE intervention has good fit with an individual's value system***	I'm one of these people, I don't listen to the people that are close to me, but if somebody from outside says it I listened and thought, hmm, do you know, I think she's right. So I changed it the next day, I cut back by about forty percent of what I was doing. (PA05)<br><br>I think because of the open and honest conversations, nothing was forced. There was never a suggestion that we must do this, or we must do that, it was a, "How do you think about trying this? What do you think …(PA08)<br><br>I'm due to retire in May, I'm 61, I've done my bit and stuff, but actually I didn't want to – I wanted to get back to work because I didn't want to sort of feel – and have to be retired on disability grounds and stuff, so it was important to me to get back to normal and then retire on my own circumstances, rather than it be forced upon me. So that has really helped me to get there really. (PA14)	For me as a therapist, I think it's been a really rewarding experience. You see that people have had discussions with me and have self-managed their own return to work through those discussions, and also things that I think are common knowledge are not necessarily so. (TA07)<br><br>I've really enjoyed it. It's been a lovely opportunity to be able to see people from the acute through to the community, to have that year follow-up and to be able to see what a difference can be made by having someone who is there throughout. (TA09)<br><br>But at the core of it it's work where it feels like a privilege to be alongside people at this real challenging time but a time when you can see a lot of growth for people (TA11)	I think (sighs) it's been difficult for me as a line manager, and I think it's been difficult for [PA08] because [PA08] has seen so many people, and of course from a company point of view, we have to protect the company as well. (EMP03)<br><br>It's an us and them situation between employers and employees, because you're trying to create a balance between the two. And so what you have to do is keep the lines of communication going, and that's absolutely key in any programme, if you're encouraging the employee to keep in contact with their employer before their employer is knocking on their door, that is so important. (EMP04)<br><br>And also protecting us as an organisation to ensure that we're doing the right thing for that individual and employee HR needs, but also from the employees lens that it's not just a line manager who doesn't have medical experience making decisions you're making informed decisions through medical professionals to make sure that both the individual and the organisational needs are met rather than it being weighted to one side. (EMP06)

Acceptability Analysis data examples by domain and participant group

Acceptability domain	Patients voice	Therapist voice	Employer voice
**Intervention coherence**<br><br>***The extent to which the participant understands the intervention and how it works***	I'm hoping to go back to work soon, so I never thought that would happen, eventually, I was just thinking I have to look for another job, and then I was thinking, well what job can I look for, because I don't know what I'm capable of doing, the OT helped me to reconcile. So no it's been so great and I'm so grateful for the opportunity to have participated, because otherwise my life would be very, very different. (PA02)<br><br>I could let off those difficult thoughts so that I could then proceeds, so it was very different from that, you know, very gently letting me go forward rather than backwards, so I always looked forward to it. I can't say – when I spoke to the psychologist I was very low so I don't think – looking forward is the wrong word, but it sort of felt necessary and helpful. (PA06)<br><br>I don't think I would have coped as well as I did without that support, I don't think I would have been able to go by myself back in work at the speed I was, and to the extent I was, without that support (PA13)	Some people have – they've all got different sort of injuries, some more severe than others, and also their workplaces vary quite a lot in terms of whether someone has support or hasn't got support or whether an employer is flexible or not or will allow reasonable adjustments or not. And some of them are very, very flexible and the injuries aren't that severe and they're kind of easily going back without much input, and others needing a lot of input so it's, yeah, very variable. (TA02)<br><br>I think the ROWTATE therapist suggesting, have you spoken to your workplace, are they aware of this injury that has happened to you, etcetera, is really valuable. Because it seems a lot easier to return someone to an original job that they've got and some workplaces, if they've got a clear understanding of where things are going, are more likely to keep a job open for a longer period of time, and/or they would like to make adjustments but they don't know what they are. (TA07)<br><br>I would say most of my participants, the intervention worked well, and they really appreciated the extra support I think. The fact it was extra on top of what they were getting from whatever usual care was, which often was very minimal, so yeah, I think generally the majority of people felt, I felt, it was a beneficial outcome (TA15)	As an employer, we have benefited from the service that ROWTATE provides with the unfortunate circumstances that other key employees, not just at a - not run of the mill, you know what I mean, not just a number, one of our senior leadership team that is responsible for driving the future of our organisation, and the support that she has had, and I received in terms of that return to work process, certainly, well, it's been a success frankly, because she's still here now and back to full time and enjoying herself, or at least she tells me she is. So that is a success and honestly, in January, February time, this time last year, I couldn't have hand on my heart have said that was going to be the case, because of course you start to have doubts when someone has been off for that period of time. So, I would give it my support (EMP01)<br><br>We'd always want to get colleagues back into work in the appropriate times and keeping them safe and well. But equally, I think the longer that the employee is off, it's much harder to get them back into work, so having that support and intervention at the right time is good (EMP05)<br><br>I think it was the very pragmatic advice of where the boundaries should lie. (EMP06)

Acceptability Analysis data examples by domain and participant group

**Opportunity Cost**<br><br>***The extent to which profits, benefits or values must be given up to engage in the intervention***	I think it would've been a bit more tiring as well if it was having to go to a physical location, because you would have to get ready, remember you've got to be somewhere, you've got to plan your journey, you've got to plan getting home, so that would be quite overwhelming initially I think. So, but for me it worked really, really, really well. (PA02)<br><br>But I wouldn't even label it as a cost really because it's not like you don't get a benefit from it. So I wouldn't call it a cost. Yes, it does take a bit of time, but it's not like you're not receiving something from it. It's not as though it's a study where you don't gain a benefit, it's just research sort of thing, you do get an output as well. (PA03)<br><br>The clinical psychology that we get through ROWTATE, that was obviously during office hours and my manager was really happy for me to take time out of work to do physio, to do psychology, anything that I needed, I could take work time to do that. (PA12)	To be honest, it's probably easier to use my own things, because (laughs) my experience of the NHS when they send you a laptop or they try and get you to sign in, again, the administrative chaos in having that is just too much. So it's actually more efficient that I just use my own things because I know how they work (TA01)<br><br>You can fit people in much more easily. And I think I'd imagine a lot of the participants appreciate it because they haven't had to travel with all the other appointments they've got going on and, you know, the travel's probably quite a challenge for them. It's actually quite nice for a good chunk of them anyway to not have go anywhere. (TA02)<br><br>I think, well the amount of contact you can have with the participants because you've always got that to factor in, the amount of time that it'll take to do the admin side of things as well. (TA03)	It was a bit a stuttering start because there was some uncertainty about how severe was it, and then as [PA02] went off sick, it became clear that actually, we were going to have a gap, and probably one of the most difficult parts for me was not so much picking up the role, which was difficult but understanding the time frame, because it went from October, okay, signed off sick through November, December, and I think she eventually came back in March, which when you talk about it in those terms, you go okay, three or four months, but when you're in the thick of it, it was a real gap to us. (EMP01)<br><br>I think that the Department for Work and Pensions should have some quality data on how long people with different conditions typically take away from work before they return to work. And AI tools should be used on that data, to find some kind of typical – might be ranges, but to find some typical ranges for how long people are away from work with different conditions, and how long it is before they return to work….This should make a massive difference, because the more we can take out the unknowns, the more likely people are to engage with the system, any system that supports people to do this. (EMP02)

Acceptability Analysis data examples by domain and participant group

Acceptability domain	Patients voice	Therapist voice	Employer voice
**Perceived effectiveness**<br><br>***The extent to which the intervention is perceived as likely to achieve the purpose***	I went back on phased return at the beginning of October, and if I hadn't had that input I think I would've probably just thought two week phased return, you know, I wouldn't have done it as gradually, so that was really good, because I did need to do it as gradually as that. (PA03)<br><br>My recovery started, I'd gone back to work, she helped with a graduated return to work programme She was very careful around I think me over committing myself, obviously she's well experienced in that field, so she was able to set the level of commitment that I should be making to my company and everything else. (PA08)<br><br>She was just so helpful and so supportive; and towards the end it was more a, right let's think about how we're going to get you back to work again without being, like pressure on, I never felt pressurised to get back to work, but it was always, let's see what we can do to get you back to work. (PA11)	I've had others that have just said, "Do you know what? I don't know where I'd be if it wasn't for this service. I don't know what I'd have done. I think I might have gone back to work too early and it would have gone wrong," or they'd have said oh, I'm not sure, come back, I didn't think of that, or I'm not sure I prepared myself this well for work, so I'm getting some really, really positive feedback of people saying how effective it's been. (TA02)<br><br>When somebody says 'You're holding me' – I mean, that was his words, not mine – that is about I am that safety net, so there is somebody to come back to (TA08)<br><br>I think the amount of positives you can get from it, and the learning you can get from it in terms of improving other services to save money so you don't have people being re-admitted, or they're not having to have a sudden— Yeah, just things like pain relief. I've had people who were really close to being re-admitted because their pain was poorly managed, but because I managed to step in and get people involved at the right time, we stopped that happening. So I think it's perfectly placed to manage all those sorts of things. (TA09)	We don't have the expert knowledge of post-head injury, serious injury care. So, I think anything like that, that can support people to come back to work is a good thing. (EMP01)<br><br>It could help everybody concerned much quicker than the conventional route of, you know, just seeing your GP, seeing the consultant at the hospital, having generic occupational health in place, you know, they're out of their depth in terms of understanding the extent of the trauma and the best treatment plan. (EMP04)<br><br>I think something that is much more rounded for individuals in terms of an all-round care programme, sounds far better and comprehensive and also tailored as well, because some individuals will need that mental health support I would imagine, much more than others as well, and the family support. It's a huge impact if you've got financial loss (EMP05)

Acceptability Analysis data examples by domain and participant group

Acceptability domain	Patients voice	Therapist voice	Employer voice
**Self Efficacy**<br><br>***The participant's confidence that they can perform the behaviour/s required to participate in the intervention***	I would say I felt confident. I guess it depends on how they deliver it to you, or what your personality, or a bit of everything. No, I felt confident that I could access it. I think I probably wouldn't have had the initiative or known how to look for some of it otherwise (PA04)<br><br>I was very confident. I don't know whether she was giving me advice; I think she was more being with me where I was and it was being able to voice where I was, you know, and those difficult times. (PA07)<br><br>Very confident because they were all things that I was - I mean the other thing that I bought was some stress balls, but everything else was easy, I could just turn easily like my dog, like glass and wood, no everything I could do. (PA11)	I think that's been a huge factor in my confidence, having a mentor that is really responsive and gives really good, experienced advice. (TA06)<br><br>I think it's definitely – it's grown because I guess being on the trial for a longer period of time and getting that feedback that this is useful and seeing how it's nudged people towards maybe slightly different outcomes, you know, you could kind of see a process going one way, somebody who was possibly in a process where they were going to lose their university place and then me and the OT attended a couple of meetings and helped them to think about how they could make some reasonable adjustments there. And that led to a change and that person being able to go back in actually. So there's definitely experiences like that that build a confidence that actually – you know, I guess in terms of my own sense of self efficacy, I can be useful to people in this role. (TA12)<br><br>I think it's improved as I've had more practice at it. I don't know that I hadn't – I guess I kind of adapted into the role a bit more and sort of flexed a bit more, been a bit more relaxed and found it easier (TA14)	I'd say 90% plus confident, because it was me that did it, so therefore I know what I know, I know what I controlled. The reason I don't give it 100%, we had the guidance from [TO20] about the phased return, which we adhered to, although I had to slap [PA02]'s wrist fairly often to say leave the office, because she's not good for that. But I carried it all through to the letter and the only bit that I would say life is busy, my job is busy, so whilst I asked the question and checked in, are you okay and do you need a break and so on, we didn't have quite so many formal meetings where we sat down and said right, you are eight weeks into a twelve week return, what has gone well, what hasn't gone well? (Emp01)<br><br>I think the only thing I would say is around policy and procedure. Again, I've been quite straightforward with our HR team. I'm not sure the right level of guidance is out there for people out there writing sickness absence management policies or flexible working policies. (Emp03)<br><br>You can do the basics of like here's a policy, but doesn't really set you up for actually managing that situation. (EMP06)
