# Supplementary material for: Stakeholder acceptability of the ROWTATE vocational rehabilitation intervention in England: an interview study
Source: BMJ Open. 2025 Oct 23;15(10):e098048. doi: 10.1136/bmjopen-2024-098048 (PMC12557785; doi:10.1136/bmjopen-2024-098048)
Supplement: Supplementary file 2 [file bmjopen-2024-098048.reviewer_comments.pdf]

PEER REVIEW HISTORY

BMJ Open publishes all reviews undertaken for accepted manuscripts. Reviewers are asked to complete a checklist review form and are provided with free text boxes to elaborate on their assessment. These free text comments are reproduced below.

ARTICLE DETAILS

Title (Provisional)

Stakeholder acceptability of the ROWTATE vocational rehabilitation intervention: An interview study

Authors

Mann, Claire; Lindley, Rebecca; Kendrick, Denise; Radford, Kathryn A; Holmes, Jain; Kellezi, Blerina; dasNair, Roshan; Fallon, Steve; Timmons, Stephen

---

VERSION 1 - REVIEW

Reviewer 1

Name Bourke, John

Affiliation The University of Sydney John Walsh Centre for Rehabilitation Research

Date 11-Apr-2025

COI None

This paper seeks to understand the acceptability of a vocational rehabilitation intervention for people who have experienced a severe or major traumatic injury. A range of patients, therapists, and employers were interviewed with data being mapped to the theoretical framework of acceptability (TFA), well used and respected implementation framework for exploring acceptability of interventions.

Overall, the paper presents a coherent overview of a very important topic which indeed requires usable and acceptable interventions to aid people to return to work after a traumatic injury. The paper has a number of strengths including using the TFA and the framework method to guide analysis, and including the COREQ checklist to provide transparency and rigour.

The paper could be improved through addressing a number of areas. Primarily, providing more information about the exact and discrete components of the intervention, and/or referring to papers that may explain this in more detail. In addition, more information is required in the methods sections expanding on how the framework method was operationalised, and potentially including some construct definition in the results would strengthen the paper.

Specific recommendations are outlined below.

I wish you all the best in your revisions.

Introduction

- Please provide more information about the specific content, scope, and delivery of the ROWTATE. This would provide the reader with a clear idea of exactly how the intervention is administered, and exactly how patients interact with ROWTATE. For example, what are the discrete components of the intervention? How regularly do patients and clinicians meet?

- You also mentioned it runs for 12 months. Do all people who participate in ROWWTATE stay with the programme that long?

-

Methods

- Pg. 6, line 28 – Is SF meant to be ST as per the names in the author list? Also, can you please explain what a trained patient and public group member is exactly?

- Please include interview schedule in supplementary documents.

- I suspect some of the references a slightly out of order. For example, does "Information power" on pg. 6, line 11, need to reference Malterud et al. (#20) not Kettlewell et al (#19)?

- Likewise, on pg. 6, line 47, Gale et al (ref #21) is the correct reference for the framework method.

- Data analysis. Please include some more information about Gale's framework method, e.g., did you use a table of theme comparison. It appears you have through looking at the analysis data examples table. However a sentence or two in the methods to explain your process here would be beneficial.

Results

- I feel it would be beneficial to provide a very brief definition (e.g. a sentence or two) of each TFA domain in the results section. Refer to the authors of the TFA for definitions. This would help to clarify the exact nature of each construct being explored by the qualitative data, and strengthen the link between your data and the construct.

- Page 11, line 27. Perhaps the word 'sustainable' would be more appropriate than 'staying there', as the idea seems to be around deeper understanding and long term flow at work.

Discussion

- Consider removing the 'summary' subheading following discussion. Just begin first paragraph explaining or reiterating the findings of the study.

- Likewise, consider removing the 'comparisons with other literature' subheading as the discussion should naturally lead into situating your findings within the wider evidence base.

- I wonder if could elaborate on why patients displayed a lack of engagement in some instances (e.g. pg. 13, line 28). You began to elaborate on this in the results section (pg. 9, line 54 - referring to a lack of financial or social capacity). I'm concerned that suggesting that therapists found this disheartening and time consuming implicitly levels the responsibility at the patients. Perhaps exploring why patients found engagement hard in some instances might provide more opportunity to address those challenges.

- Or page 14, line 17, consider mentioning that any roll-out would require an implementation plan involving strategies and measure to assess those strategies.

**Reviewer**	**2**
**Name**	**Sousa, Luis**
**Affiliation**	**Atlantica University**
**Date**	**10-May-2025**
**COI**	**None**

The manuscript"Stakeholder acceptability of the ROWTATE vocational rehabilitation intervention: An interview study" aimed "to explore and understand the acceptability of vocational rehabilitation after traumatic injury for patients, therapists and employers". The title is clear and appropriate. The abstract is structured and appropriate.

The introduction justifies the study. The method is described comprehensively according to the SRQR guidelines. However, specific details to ensure the scientific validity of research results must be described in the research and analysis process. There is no information on how the researchers reached agreement, and additional descriptions are needed on how the researchers ensured reliability in the coding process (what the criteria are). The results are clear and adequate. The findings are contextualized with verbatim excerpts from the participants' interviews as is supposed. In the discussion, the results are presented and compared with other studies. The implications for practice, health policies and future investigations are clearly presented. The limitations of the study are presented. It is recommended to include "conclusions" where the findings are summarized in order to verify that the objective has been achieved.

VERSION 1 - AUTHOR RESPONSE

**Comments**	**Response**
**Reviewer 1**	
Introduction<br>- Please provide more information about the specific content, scope, and delivery of the ROWTATE. This would provide the reader with a clear idea of exactly how the intervention is	Thank you, we had originally included this but referenced as supplementary data, due to the word count. We have now added this back in as Table 1:

administered, and exactly how patients interact with ROWTATE. For example, what are the discrete components of the intervention? How regularly do patients and clinicians meet?	The following table outlines the key components of the intervention and it's delivery mechanisms: Table 1 (added)
- You also mentioned it runs for 12 months. Do all people who participate in ROWWTATE stay with the programme that long?	We have clarified as follows:<br>The remotely-delivered intervention was individually tailored and support was provided by occupational therapists (OTs) and, where needed, clinical psychologists (CPs) for up to 12 months dependent on need<br>We have also added an item to the table to clarify further<br>Patient is discharged when returned to work, work is stable, and no longer requires monitoring or support or at 12 months, whichever sooner.
**Methods**	
- Pg. 6, line 28 – Is SF meant to be ST as per the names in the author list?	No, SF is the PPI researcher, he was added to the author list
Also, can you please explain what a trained patient and public group member is exactly?	We have clarified as follows:<br>one patient and public involvement (PPI) group member (SF) who was trained in interviewing and jointly interviewed some patient participants alongside a researcher
- Please include interview schedule in supplementary documents.	**Added as supplementary data – S1**
- I suspect some of the references a slightly out of order. For example, does "Information power" on pg. 6, line 11, need to reference Malterud et al. (#20) not Kettlewell et al (#19)?<br>- Likewise, on pg. 6, line 47, Gale et al (ref #21) is the correct reference for the framework method.	References have been double checked and are now in the correct numbering system
- Data analysis. Please include some more information about Gale's framework method, e.g., did you use a table of theme comparison. It appears you have through looking at the analysis data examples table. However a sentence or two in the methods to explain your process here would be beneficial.	Thank you – we have added to the data analysis section: To ensure reliability, a second researcher (RL) independently double-coded 10% of the transcripts. Coding discrepancies were discussed and resolved through consensus, with agreed criteria recorded in a coding decision log. A working analytical framework was developed and applied across all transcripts. Data were then charted into a framework matrix to facilitate comparison across cases and domains
**Results**	
- I feel it would be beneficial to provide a very brief definition (e.g. a sentence or two) of each TFA domain in the results section. Refer	Thank you, we had this in originally and removed it due to the limitations of the word count.

to the authors of the TFA for definitions. This would help to clarify the exact nature of each construct being explored by the qualitative data, and strengthen the link between your data and the construct.	We have added in one line summaries under each heading e.g.<br>The affective domain considers attitudes towards the intervention.
- Page 11, line 27. Perhaps the word 'sustainable' would be more appropriate than 'staying there', as the idea seems to be around deeper understanding and long term flow at work.	Thank you, we have changed to:<br>Therapists perceived ROWTATE as effective in RTW and sustaining employment.
**Discussion**	
- Consider removing the 'summary' subheading following discussion. Just begin first paragraph explaining or reiterating the findings of the study.<br>- Likewise, consider removing the 'comparisons with other literature' subheading as the discussion should naturally lead into situating your findings within the wider evidence base.	We used these subheadings to conform with the journal guidelines. We have therefore not made these changes.
- I wonder if could elaborate on why patients displayed a lack of engagement in some instances (e.g. pg. 13, line 28). You began to elaborate on this in the results section (pg. 9, line 54 - referring to a lack of financial or social capacity). I'm concerned that suggesting that therapists found this disheartening and time consuming implicitly levels the responsibility at the patients. Perhaps exploring why patients found engagement hard in some instances might provide more opportunity to address those challenges.	We agree this is an area of interest for further and have added a small explanation (within the limitations of the word count)<br>Disengagement could be in part due to a recruitment of patients who did not need this specialised support (rapidly healing injuries, lack of cognitive injuries). We are exploring this further as part of broader trial evaluation.<br><br>We have also moved the sentences around to remove the implication that the patients are responsible:<br>Both therapists and patients understood ROWTATE filled a gap in usual care. Lack of engagement was disheartening and time consuming for therapists.<br>Some patients did not have financial or social capacity to engage or did not engage because they felt they did not need support.
- Or page 14, line 17, consider mentioning that any roll-out would require an implementation plan involving strategies and measure to assess those strategies.	Thank you – we have added to the implications:<br>Roll-out would require an implementation plan involving strategies and evaluation measures to assess those strategies.
**Reviewer 2**	
specific details to ensure the scientific validity of research results must be described in the research and analysis process. There is no information on how the researchers reached agreement, and additional	**As above:** Thank you – we have added to the data analysis section: To ensure reliability, a second researcher (RL) independently double-coded 10% of the transcripts. Coding discrepancies were discussed and resolved

descriptions are needed on how the researchers ensured reliability in the coding process (what the criteria are).	through consensus, with agreed criteria recorded in a coding decision log. A working analytical framework was developed and applied across all transcripts. Data were then charted into a framework matrix to facilitate comparison across cases and domains
It is recommended to include "conclusions" where the findings are summarized in order to verify that the objective has been achieved.	We have added<br>**Conclusions**<br>A vocational rehabilitation intervention delivered remotely by OTs and CPs is acceptable to patients, therapists and employers.
